# Unexpected Findings on Histology: Plant Seeds Inducing and Mimicking Gastrointestinal Diseases

**DOI:** 10.3390/diagnostics16060826

**Published:** 2026-03-10

**Authors:** Fanni Hegedűs, Tamás Lantos, Anita Sejben

**Affiliations:** 1 Department of Pathology, University of Szeged, 6725 Szeged, Hungary; 2 Department of Medical Physics and Informatics, University of Szeged, 6720 Szeged, Hungary

**Keywords:** plant seed, phytobezoar, colon polyp, acute appendicitis

## Abstract

Foreign material is an uncommon finding in routine gastrointestinal histopathology, but may occasionally contribute to disease pathogenesis or create diagnostic pitfalls. We report two illustrative cases highlighting the diverse clinical and histologic implications of ingested plant material. The first case involves a 10-year-old boy who presented with clinical features consistent with acute appendicitis and underwent appendectomy. Although gross examination revealed a macroscopically unremarkable appendix, histological evaluation demonstrated mucosal ulceration associated with an impacted plant seed within the appendiceal lumen, supporting a diagnosis of obstructive acute appendicitis. The second case describes a 60-year-old woman undergoing a screening colonoscopy, during which a small sessile lesion in the transverse colon was resected. Histologic examination revealed no colonic mucosa; instead, the specimen consisted entirely of plant material, morphologically consistent with a tomato seed, representing an incidental finding mimicking a colonic polyp. These cases underscore that plant seeds, while rare, may act as obstructive agents in appendicitis or simulate true pathological lesions during endoscopic and histologic evaluation. Awareness of the characteristic microscopic features of plant material is essential to avoid misdiagnosis and to recognise their potential clinical and forensic relevance.

**Figure 1:**
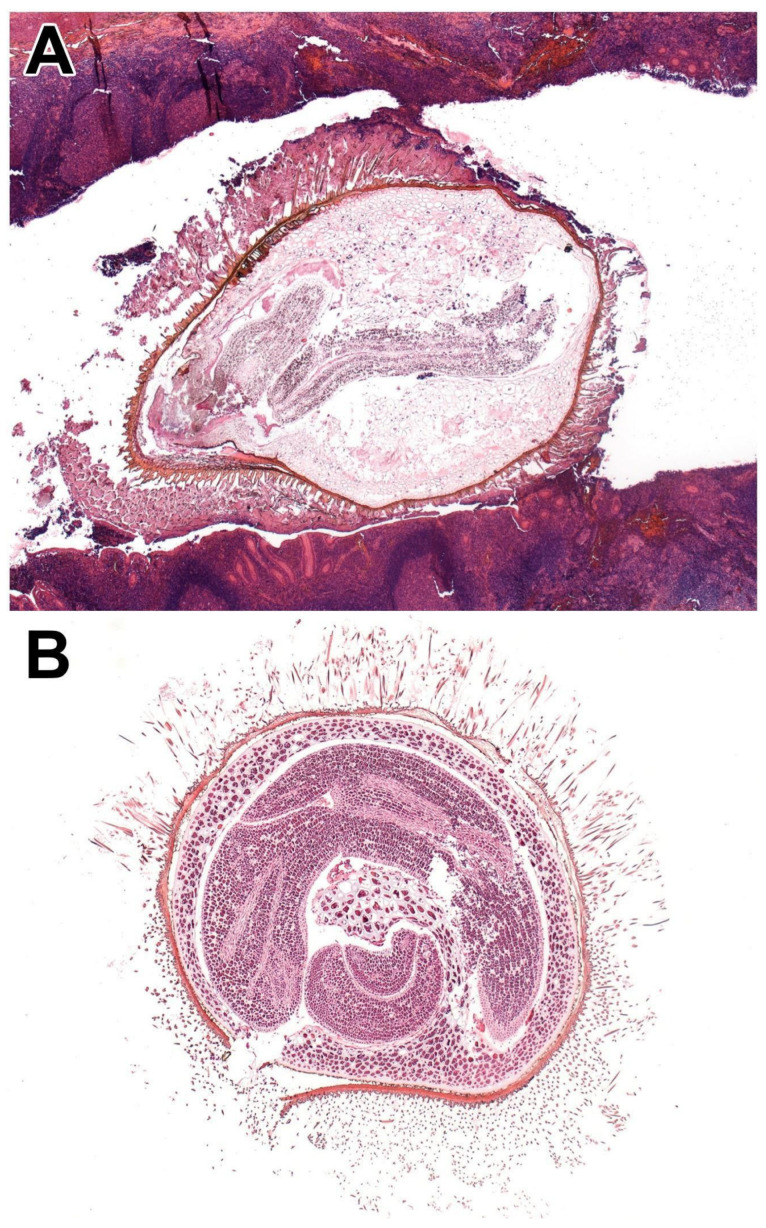
A 10-year-old boy presented with fever, vomiting, and severe right lower quadrant abdominal pain. An abdominal ultrasound demonstrated free intraperitoneal fluid; although the appendix was not visualised, the clinical presentation was considered consistent with acute appendicitis. Consequently, an appendectomy was performed. Gross examination revealed an appendix of normal calibre without macroscopic evidence of perforation or serosal inflammation. However, sectioning of the distal appendix identified a firm intraluminal, calculus-like structure measuring up to 3 mm in greatest dimension, associated with focal mucosal hyperemia. Histological examination of hematoxylin and eosin (HE)-stained sections demonstrated mucosal ulceration, transmural acute inflammation, early signs of peritonitis, and an impacted plant seed within the appendiceal lumen, indicative of acute appendicitis (**A**). A 60-year-old woman with no significant past medical history underwent a screening colonoscopy. Endoscopic examination demonstrated preserved haustration of the cecum, ascending colon, hepatic and splenic flexures, descending colon, and rectum. In the transverse colon, a sessile polyp embedded in mucus measuring up to 5 mm in greatest dimension was identified; therefore, polypectomy was performed. Following complete tissue embedding and sectioning at multiple levels until no further tissue remained, HE sections revealed no colonic mucosa; instead, the specimen consisted exclusively of plant material (**B**). Based on the morphological criteria described by Campora et al. [1], the foreign material was identified as a tomato seed, characterised by a central embryo surrounded by an outer seed coat with associated filamentous structures. This finding had no clinical significance and was regarded as an incidental and noteworthy histologic observation that might draw attention to the fact that food remnants might mimic colon polyps via endoscopy. Gastrointestinal specimens constitute a substantial proportion of routine histopathological workload; however, the presence of foreign material is uncommon in routine gastrointestinal biopsy samples. Nevertheless, in a limited number of cases, plant seeds or other plant-derived particles may be encountered. The prevalence of such findings may rise as vegetarian diets become more common. Foreign bodies are a recognised contributing factor in the pathogenesis of acute appendicitis, with plant seeds reported as the third most frequently identified intraluminal foreign body. The presence of plant seeds and remnants in gastrointestinal specimens has been sporadically reported in the literature. Most series are descriptive or case-based, and systematic epidemiological studies are few. In appendectomy specimens, incidental plant material is variably observed, but available case series suggest that true seed-associated appendicitis is rare compared with obstructive agents. Once a foreign object enters the appendiceal lumen, retrograde passage into the colon is not possible, resulting in luminal obstruction, increase in intraluminal pressure, venous congestion, ischemic injury of the appendiceal wall, and secondary bacterial overgrowth. Common causes of luminal obstruction include fecaliths, parasitic infestation (e.g., *Enterobius vermicularis*), lymphoid hyperplasia, and appendiceal neoplasms, which may constitute a clinical diagnostic challenge, particularly when determining the underlying cause of appendicitis or intestinal obstruction [2,3]. However, luminal obstruction alone does not invariably lead to appendicitis; patients may present with clinical features mimicking acute appendicitis, ultimately leading to an appendectomy despite the absence of overt inflammatory changes [4,5]. In addition to luminal obstruction, other etiological factors implicated in the development of appendicitis include an elongated appendix, abdominal trauma, hypersensitivity reactions, appendiceal diverticulosis, and acute mesenteric ischemia [6,7,8,9,10,11]. Foreign materials may mimic true pathological lesions, such as polyps, thereby creating diagnostic pitfalls in both clinical and histological evaluation. Recently, with the advancements in next-generation endoscopic equipment, it is easier for endoscopists to identify whether a polyp is part of the mucosa, or is likely a foreign body. In addition, ingested plant material may have forensic relevance in medicolegal investigations, including cases of homicide or suicide, as seeds can preserve characteristic morphologic features during digestion and thus provide information regarding dietary intake prior to death [12]. In some cases, regionally specific or seasonal plant taxa may contribute associative evidence linking an individual to a particular location or environment. Microscopically, plant material typically exhibits polygonal architecture, thick cellulose-rich cell walls, and intracytoplasmic starch granules; distinctive pigmentation or other characteristic features may also be present. Depending on their biological impact, retained plant remnants can elicit a local foreign-body-type inflammatory reaction. These materials can occasionally mimic parasitic elements, such as *Enterobius vermicularis* eggs or larvae, or other structures including fungal hyphae, vegetable fibres, or mucus plugs. Awareness of these distinguishing features is essential, particularly when plant material is embedded within the mucosa, to reduce the risk of misdiagnosis. Recognition may be challenging given the diversity of morphologies and the limited literature describing their histologic appearance [13,14]. Previous studies, including Campora et al. and the systematic review by Razzano et al., have characterised commonly encountered gastrointestinal seeds and fruit or vegetable particles [1,15]. Unusual obstructive agents such as needles, pins, and even bullets have also been described in the literature. Sharp, pointed, and elongated foreign bodies are associated with a higher risk of perforation, whereas seeds are considered to carry a moderate risk for this complication [2]. The earliest report dates back to 1816, when Prescott et al. described a 42-year-old man who presented with abdominal pain and subsequently died; autopsy revealed acute purulent peritonitis and cocoa or chocolate nut seeds obstructing the appendiceal orifice [16]. Velanovich et al. described a vegetable seed simulating a colonic polyp in a 54-year-old woman during follow-up colonoscopy [17]. Taj et al. reported an intramucosal plant residuum detected during colonoscopy in a 27-year-old woman with chronic constipation and a family history of colorectal cancer [18]. Besides luminal obstruction or inflammation, in rare cases, plant seeds may contribute to the formation of phytobezoars as well [11]. Phytobezoars result from the incomplete digestion of plant material and seeds and may cause obstruction at any level of the gastrointestinal tract [19,20,21,22,23,24,25,26,27]. Foreign bodies, parasites, and plant seeds are most commonly identified incidentally during the histopathological examination of appendectomy specimens or resected large bowel samples. However, impacted seeds may contribute to the development of obstructive appendicitis or mimic colorectal polyps. Plant seeds exhibit considerable morphologic variability; while some can be readily recognised, others may pose differential diagnostic challenges due to their resemblance to parasitic structures, potentially leading to misinterpretation. In cases of potential clinical misdiagnosis, a comprehensive histological evaluation is warranted, including the examination of additional deeper tissue sections to ensure accurate assessment.

## Data Availability

The original contributions presented in this study are included in the article. Further inquiries can be directed to the corresponding author.

## Abbreviation

The following abbreviation is used in this manuscript:

HE	Hematoxylin and eosin
